# Volatile Terpenes and Brain Function: Investigation of the Cognitive and Mood Effects of *Mentha* × *Piperita* L. Essential Oil with In Vitro Properties Relevant to Central Nervous System Function

**DOI:** 10.3390/nu10081029

**Published:** 2018-08-07

**Authors:** David Kennedy, Edward Okello, Paul Chazot, Melanie-Jayne Howes, Samuel Ohiomokhare, Philippa Jackson, Crystal Haskell-Ramsay, Julie Khan, Joanne Forster, Emma Wightman

**Affiliations:** 1 Brain, Performance and Nutrition Research Centre, Northumbria University, Newcastle upon Tyne NE1 8ST, UK; philippa.jackson@northumbria.ac.uk (P.J.); crystal.haskell-ramsay@northumbria.ac.uk (C.H.-R.); julie.khan@northumbria.ac.uk (J.K.); jo.forster@northumbria.ac.uk (J.F.); emma.l.wightman@northumbria.ac.uk (E.W.); 2 Human Nutrition Research Centre, Institute of Cellular Medicine, Medical School, Newcastle University, Newcastle upon Tyne NE2 4HH, UK; edward.okello@ncl.ac.uk; 3 Department of Biosciences, Durham University, Durham DH1 3LE, UK; paul.chazot@durham.ac.uk (P.C.); samuel.ohiomokhare@durham.ac.uk (S.O.); 4 Natural Capital and Plant Health Department, Royal Botanic Gardens Kew, Jodrell Laboratory, Richmond TW9 3AB, UK; M.Howes@kew.org

**Keywords:** mint, *Mentha*, terpenes, cognition

## Abstract

**Background:** Extracts of several members of the monoterpene-rich Lamiaceae sub-family Nepetoideae, including those from the *Salvia* (sage), *Melissa* (*Lemon balm*) and *Rosmarinus* (rosemary) genera, evince cognitive and mood effects in humans that are potentially related to their effects on cholinergic and GABAergic neurotransmission. To date, despite promising in vitro properties, the cognitive and mood effects of the closely related *Mentha spicata* (spearmint) and *Mentha piperita* (peppermint) remain unexplored. This study therefore assessed the human cognitive/mood effects of the *M. spicata/piperita* essential oil with the most promising, brain-relevant in vitro properties according to pre-trial in vitro screening. **Design:** Organic spearmint and peppermint (*Mentha spicata/piperita*) essential oils were pre-screened for neurotransmitter receptor binding and acetylcholinesterase (AChE) inhibition. In a double-blind, placebo-controlled, balanced cross-over study, 24 participants (mean age 25.2 years) consumed single doses of encapsulated placebo and 50 µL and 100 µL of the most promising essential oil (peppermint with nicotinic/GABAA receptor binding and AChE inhibitory properties, that increased calcium influx in a CAD cell neuronal model). Psychological functioning was assessed with mood scales and a range of standardised, cognitively demanding tasks pre-dose and at 1, 3 and 6 h post-dose. **Results:** The highest (100 µL) dose of essential oil improved performance on the cognitively demanding Rapid Visual Information Processing task (RVIP) at 1 h and 3 h post-dose and both doses attenuated fatigue and improved performance of the Serial 3 s subtraction task at 3 h post-dose. **Conclusion:** Peppermint (*Mentha piperita*) essential oil with high levels of menthol/menthone and characteristic in vitro cholinergic inhibitory, calcium regulatory and GABA_A_/nicotinic receptor binding properties, beneficially modulated performance on demanding cognitive tasks and attenuated the increase in mental fatigue associated with extended cognitive task performance in healthy adults. Future investigations should consider investigating higher doses.

## 1. Introduction

The Lamiaceae, or mint family of flowering plants, comprises over 7000 plant species. Its largest sub-family, the Nepetoideae, comprises numerous species, including those in the genera *Origanum* (oregano), *Ocimum* (basil), *Thymus* (thyme), *Salvia* (sage), *Lavandula* (lavender), *Melissa* (*Lemon balm*), *Rosmarinus* (rosemary) and *Mentha* (mint; e.g., spearmint (*M. spicata* L.) and peppermint (*M. piperita* L.)). Plants in these genera typically synthesize high concentrations of volatile and pleasantly odorous mono-/sesqui-terpenes which include menthol, menthone, menthofuran, isomenthone, (*E*)-caryophyllene, 1,8-cineole, linalool, limonene, carvone, pulegone and α-terpineol [1]. Consequently, this plant group is a particularly rich source of essential oils (usually a steam-distilled liquid containing volatile aroma compounds). Evidence suggests that the psychotropic properties of the above plants reside in these volatile constituents and, as a consequence, the essential oils they produce may also exert neurocognitive effects.

As an example, in vitro studies show that *Lemon balm* (*Melissa officinalis* L.) essential oil has an affinity for gamma-aminobutyric acid A (GABA_A_) receptors and exert a net depressant effect on neuronal activity (commensurate with anxiolytic effects) as assessed by patch clamp electrophysiology [2]. Several randomised, double-blind, placebo-controlled, balanced-crossover trials have also demonstrated anxiolytic-like effects in humans following single doses of ethanolic extract or dried leaf powder of *Lemon balm* [3,4,5]; whilst chemically-characterised and combined ethanolic extracts of sage (*S. officinalis* L.), rosemary (*Rosmarinus officinalis* L.; accepted name: *Salvia rosmarinus* Schleid.) and *Lemon balm* improved memory in a randomised, double-blind, placebo-controlled pilot trial [6]. Similarly, single doses of dried leaf [7] and an ethanolic extract [8] of sage; containing a wide spectrum of phytochemicals, have both been shown to significantly improve mood and cognitive function. However, the most recent of these studies confirmed that the psychoactive properties of sage were evident following an essential oil composed exclusively of monoterpenes. This and other essential oils, as well as a wide range of sage extracts, also exhibited in vitro cholinesterase inhibiting properties [7,9,10,11,12,13,14,15,16], that were potentially predicated on synergies between the monoterpene components [12,13].

To date, with the exception of consistent evidence of cognitive/mood effects for sage, *Lemon balm* and rosemary, the psychoactive potential of many of the remaining edible aromatic Lamiaceae species remain under-investigated. One of the most promising groups of plants in this respect is the *Mentha* genus and, in particular, *Mentha spicata* (spearmint) and *Mentha* × *piperita* (peppermint); which are widely used as food components, flavourings and for their medicinal properties. The latter include uses as antispasmodics [17], anti-emetics [18] and treatments for irritable bowel syndrome and as topical analgesics [19].

Members of the genus typically express several monoterpenes that provide their “minty” flavour and odour; most notably menthol, menthone, limonene, piperitone, carvone and their derivatives, alongside the more typical monoterpenes expressed by related genera [20,21]. These monoterpene components may exert a number of effects directly relevant to brain function. These include: cholinesterase inhibition activity by essential oils [20,22,23]; allosteric serotonin 5-hydroxytryptamine-3 (5-HT_3_) receptor modulation by menthol alone and peppermint oil [24]; negative allosteric modulation of GABA_A_ receptors by carvone [25]; and positive allosteric modulation of GABA_A_ receptors by menthol [26,27,28]. Menthol also exerts several other unique effects which may be tangentially relevant to central nervous system (CNS) functioning; including the allosteric activation of glycine receptors [26] and modulation of the function of transient receptor potential cation channel subfamily M member 8 (TRPM8) ”cold menthol” receptors [29] and kappa opioid receptors [30].

In terms of the effects of these CNS interactions, investigations utilizing animal models have observed increased ambulatory behaviour [31], improved memory [32] and anxiolytic effects which are potentially driven by modulation of serotonergic or dopaminergic function [33]. Currently, only one controlled trial has investigated the brain function effects of *Mentha* species in humans. Here promising effects were observed on quality of working memory and spatial working memory as well as improved sleep onset and vigour, alertness and overall mood in a group of 50–70 years olds following 90 days supplementation with 600 mg and 900 mg of an aqueous extract of *Mentha spicata* (spearmint) that contained the phenolic constituents but not the terpene constituents of the plant material [34]. The current study extends this research by investigating the cognitive and mood effects of oral administration of mint essential oil comprised of the volatile terpene phytochemical constituents of a *Mentha* plant, in healthy, young humans. A preliminary in vitro analysis assessing the acetylcholinesterase (AChE) inhibitory properties and GABA_A_, glutamate *N*-Methyl-d-aspartic acid (NMDA) and nicotinic receptor binding properties of a range of organic *M. spicata* and *M. piperita* essential oils was conducted in order to select a treatment liable to exert CNS effects relevant to cognitive function and mood.

## 2. Pre-Trial in Vitro Screening of Essential Oils

### 2.1. Methods

#### 2.1.1. Receptor Binding

Six organic *Mentha spicata* and *M. piperita* essential oils were investigated in radioligand competition binding assays using a range of ligand-gated ion channel radioligands targeting the GABA_A_, neuronal nicotinic and NMDA glutamate receptors. 

An initial screen was performed at 0.1 mg/mL for each mint essential oil versus [^3^H] flunitrazepam, [^3^H] nicotine and [^3^H] MK801 (as described in [35]). 

A series of dose–response competition binding experiments were then performed with the essential oil that exhibited the most interesting pattern of receptor binding (test concentrations, 0.0001–1 mg/mL) using [^3^H] flunitrazepam, [^3^H] MK-801 and [^3^H] nicotine, using well-washed adult rat forebrain membranes [35].

The data were fitted to a non-linear regression dose response curve with variable slope and apparent pIC_50_ values determined using GraphPad Prism 4. Apparent pIC_50_ values Vs [^3^H] flunitrazepam and [^3^H] nicotine were 1.79 ± 0.11 and 1.88 ± 0.24, respectively. No significant effect was seen on [^3^H] MK801 binding up to a concentration of 1 mg/mL (*n* = 3 independent experiments performed in triplicate)

#### 2.1.2. AChE Inhibition

The ability of the essential oils to inhibit acetylcholinesterase (AChE) from *Electrophorus electricus* was assessed using the colorimetric method of Ellman [36] as described by Okello et al. [37].

#### 2.1.3. GC-MS Analysis

The GC-MS analyses were performed using an Agilent 7890A GC coupled to an Agilent 5975C MS. The oils were analysed using the GC-MS method described previously [35]. In summary, chromatography was performed using a 30 m × 0.25 mm ID × 0.25 μm DB-5 MS column (J & W Scientific Inc., Rancho Cordova, CA, USA) using a temperature programme of 40–300 °C at a rate of 3 °C/min. The carrier gas was helium at a flow rate of 1 mL/min and the injection volume was 1 μL (split 1:10) at 220 °C, via an autosampler. Detection was by MS, fitted with electrospray ionisation source operated at 70 eV, with a source temperature of 180 °C; mass spectra were recorded in the range *m*/*z* 38–600. Compounds were identified by comparing retention indices and/or mass spectra with published data [38,39]. Percentage compositions were calculated by integrating peaks for detected oil constituents in total ion chromatograms.

#### 2.1.4. Neuronal Calcium Mobilisation

Menthol, the major constituent of *M. piperita* oil (36% in this test sample) has been previously shown to stimulate calcium mobilisation in a range of cell types, including neurons [40]. In order to establish the functional neuronal effects of the chosen essential oil neuronal calcium mobilisation was quantified in differentiated CAD cells.

A 2 mM stock of the Calcium Green™-1AM (ThermoFisher Scientific, Cramlington, UK) probe was prepared in DMSO (Sigma, Poole, UK); this was stored at −20 °C for up to 2 months. On the day of the imaging experiments the probe stock was brought to room temperature and then diluted in HEPES physiological buffer (150 mM NaCl, 1 mM MgCl_2_, 10 mM HEPES, 2 mM CaCl_2_, 5 mM KCl) to a 1 μM solution. The cells were plated in flat bottom µ-dishes (60 µ-Dish, 35 mm, high glass flat bottom, Ibidi GmbH). The cells were washed with 1 mL of physiological buffer and incubated for 40 min in 1 mL of the Calcium-Green solution (1 μM). After the incubation period, the cells were washed again with the HEPES physiological buffer and maintained in HEPES physiological buffer prior to the imaging experiments. All reagents applied to the clonal cells during the experiments were dissolved in the HEPES physiological buffer.

The incubation period for each treatment was for periods of 250 cycles (0.63 s each) before the appliance of the depolarising buffer (50 mM KCl) and then imaged for a further period of 250 cycles. All the solutions were kept at 37 °C to avoid any stress on the cells during the sample preparation and the imaging experiment. For the imaging, the Zeiss LSM 850 with an Airyscan microscope was used. The laser used for imaging was set at 514 nm: 1.1% (Ch2GaAsp: 524–620). The spectrum window was set between 24,620 and a pin hole of 49 µm was used. A size 63 oil lens with an LA of 1.4 was used to capture each image and the time between each frame (time series) was set at zero. The duration for one frame was 633.02 ms. To extract the figures and fluorescence intensities, the Zeiss software was used.

The neuroprotective properties of the selected essential oil, assessed in CAD cell cultures subjected to hydrogen peroxide were also investigated (methods and results in online Supplementary Materials).

### 2.2. Results

The GABA_A_, nicotinic and NMDA receptor binding properties, acetylcholinesterase (AChE) inhibition and major terpene constituents of the 6 organic essential oils included in the initial in vitro screening are shown in Table 1. The full GC-MS analysis results for the essential oils can be found in the online supplementary materials (Table S1). 

On the basis of high GABA_A_ and nicotinic receptor binding and AChE inhibitory properties, essential oil 4 (see Table 1) was taken forward into the dose-response experiments and the investigation of neuronal calcium mobilisation and neuroprotective properties. 

**Figure 1:**
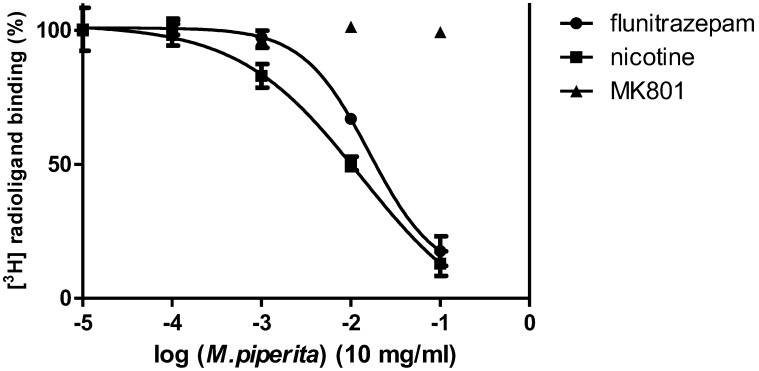
Dose-response radioligand binding properties of *M. piperita* essential oil (sample 4) in adult rat forebrain membranes at concentrations between, 0.000 and 1 mg/mL using [^3^H] flunitrazepam, [^3^H] MK-801 and [^3^H] nicotine.

In the dose–response competition binding investigation the chosen *M. piperita* essential oil inhibited both [^3^H] nicotine and [^3^H] flunitrazepam binding in a concentration-dependent manner (and with similar apparent IC_50_ values) with no significant effects on [^3^H] MK801 binding (see Figure 1.). This was consistent with previous studies focusing on menthol, the major component of *M. piperita* oil [41,42].

**Figure 2:**
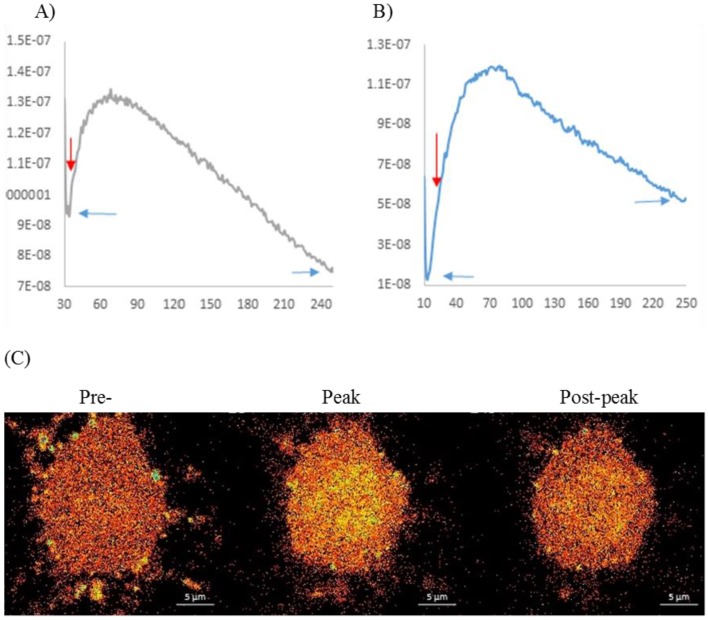
Effects of *M. piperita* essential oil on Calcium mobilisation in differentiated CAD cells. *M. piperita* essential oil (0.1 mg/mL) was applied (red arrow) and recorded for 250 cycles. Representative of at least 5 cells from 3 separate experiments. (**A**) Positive modulation on calcium intracellular mobilisation. A rapid increase in calcium was observed in the neuronal cell body, which peaked approximated cycle 40 post-application (approx. 30 s) and then dropped steadily to a level below the original starting concentration at cycle 250. (**B**) Extension of depolarization—induced calcium mobilisation. In the presence of *M. piperita* essential oil (0.1 mg/mL) for approx. 180 s, a depolarisation pulse for 250 cycles elicited a rapid calcium increase followed by a delayed decrease compared to a depolarising pulse alone, which desensitised significantly quicker. (**C**) Representative images. Representative images of individual cell body calcium changes in the presence of *M. piperita* essential oil (0.1 mg/mL), pre-application, peak of calcium increase and post peak reduction in calcium levels.

The results of the calcium mobilisation assay are presented in Figure 2.

## 3. Human Intervention Study

### 3.1. Methods

#### 3.1.1. Participants

24 participants (9 Male/15 Female, Mean age 25.2 years, Range 21–35 years) reported themselves to be in good health and free from illicit drugs, alcohol, prescription medication (apart from contraception in the case of women) and herbal extracts/food supplements at each assessment. Participants confirmed that they would also comply with these exclusion criteria for the duration of the study and that any changes in medication or health status would be reported to the researcher when they occurred. Participants who had suffered a head injury, neurological disorder or neuro-developmental disorder were excluded from participation, as were those who did not have English as their 1st language (or were not equivalent to a native English speaker; due to the cognitive tasks utilized here not being validated on non-native English speakers) had any relevant food allergies or intolerances, digestive problems, smoked tobacco, drank excessive amounts of caffeine (more than 600 mg day as assessed by a caffeine consumption questionnaire), were pregnant, seeking to become so, or were breast feeding.

The study received ethical approval from the Northumbria University Psychology department (within the faculty of Health and Life Sciences) ethics committee (code SUB058_Wightman_160216) and was conducted according to the Declaration of Helsinki (1964). All participants gave their written informed consent prior to their inclusion in the study.

#### 3.1.2. Treatments

The treatment administered in the study (essential oil number 4) was chosen from six organic essential oils on the basis of the in vitro pre-screening as described above. 

On each study day participants received two 500 μL capsules containing one of the following treatments:

100 μL *Mentha piperita* essential oil (in vegetable oil)
50 μL *Mentha piperita* essential oil (in vegetable oil)
Placebo (vegetable oil)

The individual participants’ treatments were administered double-blind in envelopes with a peppermint aroma according to the participant’s randomly allocated position on a Latin square counterbalancing the order of treatments across the cohort. Treatment guess data collected at the end of the final study visit showed that participants’ ability to identify treatments did not differ from chance. Capsules were consumed with 200 mL full fat milk (to aid distribution and absorption of lipid soluble terpenes) under constant supervision.

#### 3.1.3. Cognitive Tasks and Mood Measures

With the exception of the State-Trait Anxiety Inventory, all cognitive tasks and mood measures were delivered via the Computerised Mental Performance Assessment System (COMPASS; see: www.cognitivetesting.co.uk), a software platform for the presentation of classic and bespoke computerised cognitive tasks, with fully randomised parallel versions of each task delivered at each assessment for each individual. Tasks were presented on a laptop PC with responses made either via a four button response box, with mouse and cursor, or by the keyboard’s linear number pad. The tasks and other components of each assessment are described below in order of completion. The timelines of each assessment are shown in Figure 2. Similar selections of tasks have previously been shown to be sensitive to a number of nutritional interventions [11,43,44]. 

#### 3.1.4. State-Trait Anxiety Inventory (STAI) [45]

During the training/screening session, participants completed the entire pen and paper STAI to indicate general levels of state and trait anxiety. During the testing session participants completed the state subscale only. The STAI state subscale is a widely used instrument for measuring fluctuating levels of anxiety. The subscale contains 20 statements (e.g., “I am calm”) each with a 4-point Likert scale. Participants rate how much they feel like each statement at the time of making the response. Scores on the STAI range from 20 to 80, with higher scores representing higher levels of anxiety.

#### 3.1.5. Bond-Lader Mood Scales [46]

These mood scales have been utilised in numerous pharmacological, psychopharmacological and medical trials. These scales comprise a total of sixteen 100 mm lines anchored at either end by antonyms (e.g., “alert-drowsy,” “calm-excited”). Participants indicate their current subjective position between the antonyms on the line. Outcomes comprise three factor analysis derived scores: “Alertness”, “Calmness” and “Contentment”.

#### 3.1.6. Picture Presentation

Fifteen black-and-white photographic images of objects and outdoor and indoor scenes were presented sequentially on screen for the participant to remember at the rate of 1 every 3 s, with a stimulus duration of one second. The same set of fifteen pictures was presented to each participant in a random order.

#### 3.1.7. Face Presentation

A set of twelve passport-style photographic images of people were presented sequentially in a random order to participants. A first and last name was assigned to each photograph and presented on the screen underneath the person’s face. Stimulus duration was one second, with a 3-second inter-stimulus duration. 

#### 3.1.8. Word Presentation

A unique set of fifteen words was presented. Words were selected at random from a large bank of words derived from the MRC Psycholinguistic Database [47] and matched for word length, frequency, familiarity and concreteness. Stimulus duration was one second, as was the inter-stimulus duration.

#### 3.1.9. Immediate Word Recall

The participant was allowed 60 s to write down as many of the words as possible. The task was scored for number correct and errors.

#### 3.1.10. Numeric Working Memory

Five random digits from 1–9 were presented sequentially for the participant to hold in memory. This was followed by a series of 30 probe digits (15 targets and 15 distractors). For each, the participant indicated whether or not it had been in the original series by a simple key press. The task consisted of 3 separate trials. Accuracy (% correct) and mean reaction time (ms) were recorded.

#### 3.1.11. Corsi Blocks Task

In this task, nine identical blue squares appeared on screen in non-overlapping random positions. A set number of blocks changed colour from blue to red in a randomly generated sequence. The cursor was locked in position until the entire sequence had been presented; at which point the participants were instructed to repeat the sequence by clicking on the blocks using the mouse and cursor. The task was repeated five times at each level of difficulty. The sequence span increased from 4 upwards, until the participant could no longer correctly recall the sequence, resulting in a span measure of nonverbal working memory, calculated by averaging the level of the last five correctly completed trials.

#### 3.1.12. Choice Reaction Time (CRT)

The CRT task requires participants to indicate, by pressing the “left” or “right” response box button, the direction of the arrow presented on the computer screen. Fifty stimuli (arrows) are presented, with varying delays, taking ~2 min to complete, depending on participant reaction time. The task is scored for percentage correct responses and reaction time (ms).

#### 3.1.13. RVIP (Completed Separately and as Part of the CDB)

The RVIP task requires the participant to monitor a continuous series of single digits for targets of three consecutive odd or even numbers. The white digits are presented on the black computer screen at the rate of 100 per minute; with eight correct target strings in each minute presented in pseudo-random order. The participant responds to the detection of a target string by pressing the appropriate response button as quickly as possible. In terms of task outcomes, RVIP is scored for number of target strings correctly detected and the average reaction time (ms) for correct detections. 

#### 3.1.14. Cognitive Demand Battery (CDB)

Multiple completions of this 9 min battery of tasks reliably increase self-ratings of mental fatigue and is sensitive to many natural interventions [48,49,50,51]. Two minutes each of Serial 3 and 7 subtractions is first completed and followed immediately by 5 min of Rapid Visual Information Processing (RVIP—as described above). Mental fatigue is self-rated after each completion of the three tasks.

Serial 3 and 7 subtractions: At the start of the 2 min task a standard instruction screen informs the participant to count backwards in 3 s or 7 s as quickly and accurately as possible, using the keyboards linear number keys to enter each response. Participants are instructed verbally at the outset that if they make a mistake they should carry on subtracting from the new incorrect number with subsequent responses scored as correct in relation to the new number. To begin, a random starting number between 800 and 999 is presented on the computer screen, which is cleared by the entry of the first response. Each three-digit response was represented on screen by three asterisks which disappeared after enter was pressed to signal completion of the response. Outcomes are the total number of correct subtractions and the number of incorrect responses.

#### 3.1.15. Peg and Ball

Two configurations of wooden peg (×3) and ball (×3; blue, green and red) diagrams are displayed, on screen, with the top diagram denoting the “goal” configuration of balls on pegs. Participants must rearrange the balls on the “starting” configuration below this to match the “goal.” They must do this in the least number of moves possible. The task is scored for average thinking time (ms), average completion time (ms) and errors (total number of moves in excess of minimum required to complete all trials).

#### 3.1.16. Delayed Word Recall

Participants had 60 s to note down as many of the words from the list presented at the beginning of the task battery as they can remember. The task was scored for number correct and errors.

#### 3.1.17. Delayed Face to Name Recall

The target faces presented at the beginning of the battery were displayed on the screen one at a time. Below each face is a list of 4 forenames and a list of 4 surnames. Participants use the mouse to select the forename and surname that they think were associated with each face at the beginning of the session. The task outcomes include percentage accuracy for overall correct forenames and correct surnames and reaction time (ms).

#### 3.1.18. Delayed Picture and Word Recognition

Word and picture recognition were completed separately. In each task participants differentiate, by pressing “yes” or “no” on the response box, between the 15 target words and pictures presented at the beginning of the test battery and 15 randomly interspersed decoy words and pictures. The tasks take ~2 min to complete and are scored for percentage of correctly recognised words/pictures and reaction time (ms).

**Figure 3:**
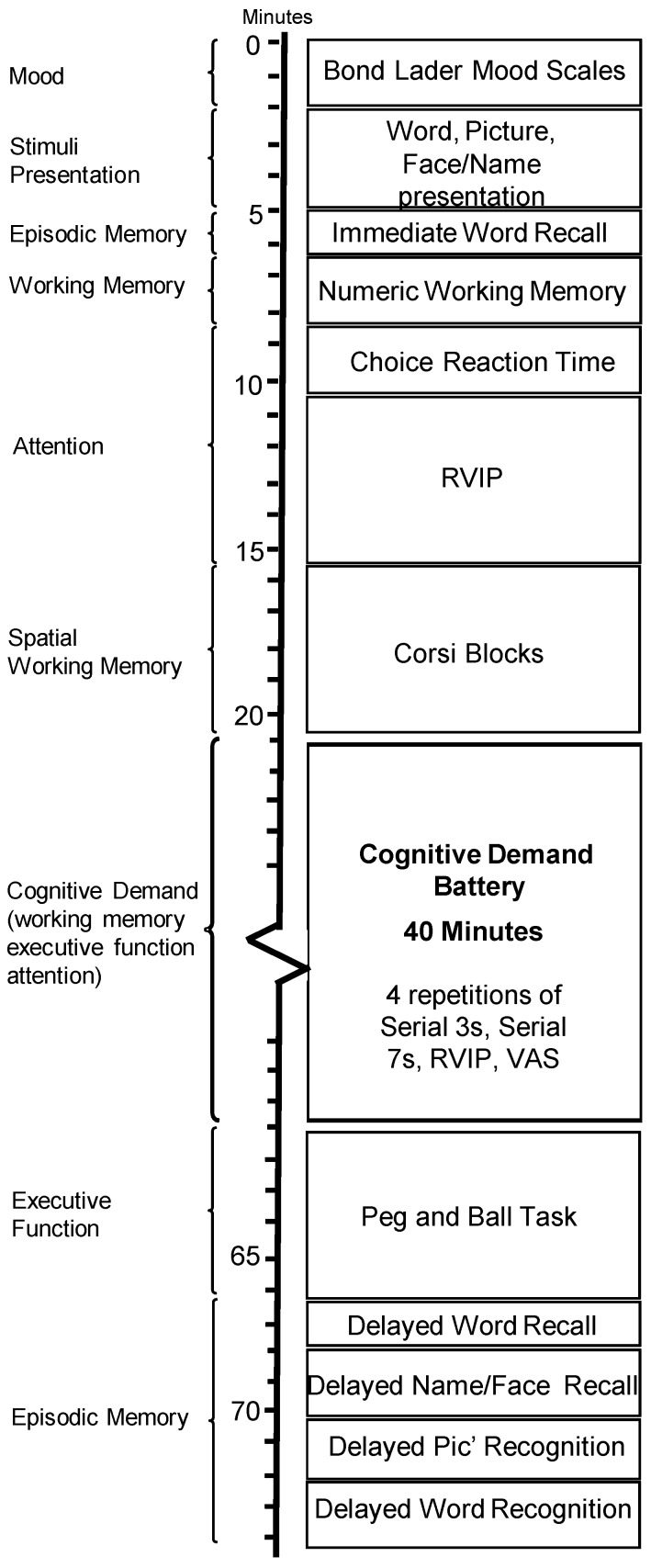
Running order of tasks administered during each assessment.

See Figure 3 for the running order of the individual cognitive assessments within the 75 min task battery.

### 3.2. Procedure

Participants were required to attend the laboratory on 4 separate occasions, the last three of which were separated by a 7 day wash-out period. The first of the visits was a training/screening session where participants gave consent and were briefed and screened against the exclusion criteria before completing several repetitions of the cognitive tasks described above to ensure they were familiar and capable of completing the tasks during the ensuing testing sessions. 

**Figure 4:**
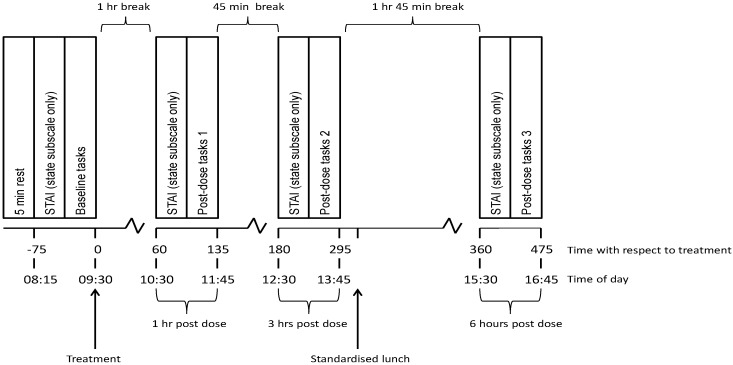
Testing session timeline for all visits.

The further 3 visits were testing sessions in which participants arrived at the testing facility at 8:00 a.m., having consumed no food for 12 h, caffeine for 18 h, alcohol for 24 h and mint-containing products for 48 h. Upon arrival, participants were screened to ensure that they still met the inclusion criteria and their light breakfast (to have been consumed before 7:30 a.m.) was noted. They then underwent a baseline cognitive and mood measurement at 8:15 a.m. Here the “state” subscale of the STAI was completed first followed by the 75 min cognitive testing battery (see Figure 3). Treatment capsules were then immediately consumed in the presence of the researcher. The cognitive assessment was then repeated commencing at 1 h, 3 h and 6 h post-dose, with a standard lunch provided after the 3 h post-dose assessment. During breaks between testing participants waited in a comfortable waiting room. The timelines of the testing day are represented in Figure 4.

### 3.3. Statistics

Statistical analyses were carried out using IBM SPSS statistics 22. Prior to analysis, task/mood data from the three post-dose assessments (1 h, 3 h, 6 h) was converted to “% change from pre-dose baseline” (Serial 3 s/7 s errors and Word Recall scores were analysed as simple numeric “change from baseline” due to the potential to score zero at baseline). In order to examine the time course of any effects of the essential oils across the testing day, the primary analysis of all task/mood data was undertaken with a priori planned comparisons, comparing data (averaged across 4 repetitions for CDB tasks) from the placebo condition to that from each of the two active treatments at each post-dose time point using *t* tests calculated using MSError from an initial omnibus within-subjects ANOVA. To reduce the possibility of Type II errors, planned comparisons are only reported for those measures that evinced a significant treatment related effect on the initial ANOVA or related multivariate test.

### 3.4. Results

Prior to the treatment code break and the analysis of cognitive task performance two participants were removed from the dataset due to inconsistent performance. 

#### 3.4.1. Cognitive Demand Battery

##### Mental Fatigue

Reference to the planned comparisons of % change from baseline data showed that subjective ratings of Mental Fatigue were significantly reduced in comparison to placebo following the higher dose of 100 μL of essential oil during the 3 h post-dose assessment (T (264) = 2.92, *p* < 0.01). (Multivariate treatment x assessment interaction (F (4, 19) = 3.56, *p* < 0.05)).

##### Rapid Visual Information Processing Task

Reference to the planned comparisons of % change from baseline data showed that, in comparison to placebo, accuracy on the RVIP task was significantly enhanced following the higher dose (100 μL) of essential oil during the 1 h (T (252) = 2.88, *p* < 0.01) and 3 h (T (252) = 2.04, *p* < 0.05) post-dose assessments. (Multivariate main effect of treatment (F (2, 20) = 3.84, *p* < 0.05)). 

##### Serial 3 s Subtractions

Reference to the planned comparisons of % change from baseline data showed that, in comparison to placebo, participants completed more correct Serial 3 s following the higher dose (100 μL) of essential oil during the 3 h (T (252) = 2.92, *p* < 0.01) post-dose assessment. (ANOVA treatment x assessment interaction (F (4, 252) = 2.64, *p* < 0.05)).

##### Serial 7 s Subtractions

Whilst there was a treatment x assessment interaction effect with regards Serial 7 s errors (ANOVA (F (4, 252) = 3.3, *p* < 0.05) this only resulted in a trend towards reduced errors following the higher dose, relative to placebo, during the 3 h assessment (T (252) = 1.9, *p* < 0.1).

**Figure 5:**
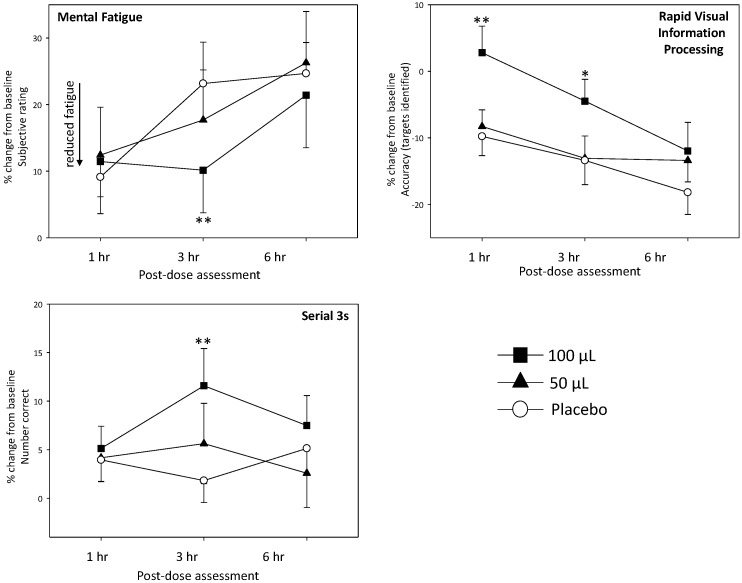
Effects of peppermint (*Mentha × piperita*) essential oil on the performance of the Cognitive Demand Battery tasks during the 1 h, 3 h and 6 h assessments. Data are mean (± SEM) % change from pre-dose baseline. Asterisks represent a significant difference to placebo from the a priori planned comparisons. *, *p* < 0.05; **, *p* < 0.01.

The measures evincing significant treatment related improvements are represented graphically in Figure 5. Data for the Cognitive Demand Battery tasks are presented in Table 2.

#### 3.4.2. Individual Cognitive Tasks

Word Recall errors was the only individual cognitive task outcome to evince a significant ANOVA result (F (4, 88) = 5.4, *p* < 0.001). However, reference to the planned comparisons showed that there were no significant differences between placebo and the low and high doses of essential oil. The data from the individual cognitive tasks are presented in the on-line Supplementary Table S2.

#### 3.4.3. Mood

There were no significant differences on the mood measures (State-Trait Anxiety Inventory—state subscale and Bond-Lader “alert,” “content” and “calm”).

The data from the mood measures are presented in the on-line Supplementary Table S3. 

## 4. Discussion

In this study, the peppermint (*Mentha* × *piperita*) essential oil selected for the human behavioural study exhibited in vitro concentration-dependent GABA_A_ and nicotinic receptor binding properties and inhibited acetylcholinesterase. When administered to human adults the higher dose of essential oil (100 μL) resulted in improvements in performance across two of the three Cognitive Demand Battery (CDB) tasks, with a trend towards improved performance on the third task. In terms of individual tasks, the accuracy of performing the RVIP task was significantly improved during the 1 h and 3 h post-dose assessments, whilst the number of correct Serial 3s subtractions was significantly increased at 3 h post-dose. This latter finding also coincided with a trend towards improved accuracy on the Serial 7s task and a significant attenuation of the subjective mental fatigue experienced by the placebo group at the same time point. Performance on the individual cognitive tasks (i.e., those only repeated once per assessment) and measurements of anxiety (STAI-state) and psychological state (Bond-Lader) were not modulated by the treatment.

Naturally, this pattern of effects raises the question as to why performance of the CDB was preferentially affected. The underlying rationale for this approach to testing, whereby participants engage in an extended period of demanding task performance, is that the increase in neuronal or psychological demands provides a more sensitive background against which to measure treatment related effects. This may be due either to the rate-limiting depletion of physiological brain resources during extended task performance, or alternatively, the increased level of engagement required by the participants. Certainly, this approach has proved sensitive to the cognitive/mental fatigue effects of a wide range of nutritional interventions [11,50,52]. An alternative explanation might be that the CDB involves four repetitions of each task (i.e., 4 times as much data), thereby increasing the statistical power of these measures to detect treatment related effects. 

In terms of in vitro properties, all of the spearmint/peppermint oils were confirmed to have the expected terpene constituents (as defined in the British Pharmacopoeia (www.pharmacopoeia.co.uk), with the spearmints typified by high levels of limonene and carvone, while the peppermints contained high levels of menthol and menthone (see Table 1). The essential oil chosen for the behavioural study had the highest level of menthol (36.7%) and it exhibited both concentration dependent GABA_A_ and nicotinic receptor binding properties, alongside significant inhibition of acetylcholinesterase (thereby potentially increasing the synaptic availability of acetylcholine). The GABAergic and cholinesterase inhibitory effects are broadly in line with previous investigations of *Mentha* × *piperita* [42] and other members of the *Mentha* genus [53]. With regards the nicotinic receptor binding, whilst there is a dearth of research assessing the effects of *M. piperita* extracts per se, menthol has been shown to modulate nicotinic receptor populations and sensitivity and inhibit nicotinic receptor mediated activity [41,54]. Similar observations have been noted with the essential oil from another member of the Lamiaceae, *Melissa officinalis* (Chazot, unpublished). In the current study, the post-hoc investigation of calcium mobilisation in a CAD cell neuronal model showed an increase in activity following application of the essential oil. It seems likely that the effects here were predicated on cholinergic upregulation, rather than any aspect of GABAergic modulation. Indeed, the behavioural consequences of increased GABAergic activity (which is inhibitory) would be expected to include modulation of mood and anxiolytic properties and potential selective impairments to cognitive function. Here, the improved performance on the Cognitive Demand Battery; which requires considerable attentional resources, would fit well with the theory of upregulated cholinergic activity. The disjunction between observations of in vitro downregulation of nicotinic receptor mediated activity by menthol [41,54] and potential cholinergic improvements in cognitive function and mental fatigue following the essential oil could be explained by several factors. First, the effects here could have been predicated on synergistic effects between component terpenes; with menthol contributing to, rather than driving the effects of the essential oil. Such synergistic effects have been seen previously with regards, for instance, AChE inhibition [12,13]. Second and similarly, the effects may have represented the balance between menthol’s receptor mediated downregulation of nicotinic activity and the essential oil’s AChE inhibition related upregulation of acetylcholine levels. Third, in vitro effects are typically measured within a short period of application and the behavioural effects here may be predicated on later effects that are different in nature, including a potential physiological “rebound” triggered by the immediate effects on cellular function. Fourth, the cholinergic effects of menthol may differ depending on the nature of the neural tissue (e.g., downregulation has typically been shown in sensory neurons). Peppermint (*Mentha* × *piperita*) essential oil also elicited an increase in [Ca^2+^] and an extension of the depolarisation response as previously reported for menthol; a major constituent of the oil [55]. The neuroprotective properties in the face of oxidative stress displayed by *M. piperita* in CAD cells (online Supplementary Appecndix I) is consistent with a previous study with a related cell line [42]. These pharmacological functional effects may also contribute, in part, to the positive cognitive effects of *M. piperita*. Of course, the net effects of the essential oil may be a result of a combination of these factors or, indeed, a host of other mechanisms of action that were not addressed in this study.

Interestingly, a previous study that assessed the cognitive effects of 50 μL of essential oil from sage (*Salvia lavandulaefolia* Vahl.; synonym for *Salvia officinalis* subsp. *lavandulifolia* (Vahl) Gams), which is in the same sub-family as *M. piperita* and synthesises many similar monoterpenes, demonstrated decreased mental fatigue and improved Serial 3 s subtractions using the same Cognitive Demand Battery [11]. As here, the effects were greater at a later time point (4 h post-dose) than at 1 h post-dose. In the previous sage study the cognitive effects also extended to the other individual cognitive tasks and included improved alertness and memory. The lower dose for the current study was selected on the basis of this previous study and given that the lower dose (50 μL) administered here was less effective, it seems likely that 100 μL of *M. piperita* essential oil may represent the lower reaches of this oil’s dose-response profile. If this is the case, higher doses of this oil may well result in a more comprehensive pattern of cognitive/mood effects. This possibility, alongside research disentangling the behavioural effects of the major components of these essential oils, deserves further research attention.

In conclusion, a peppermint (*M. piperita*) essential oil with high levels of menthol and menthone and characteristic in vitro AChE inhibitory, calcium regulatory, GABA_A_ receptor and nicotinic receptor binding properties, beneficially modulated performance of demanding cognitive tasks and attenuated the increase in mental fatigue associated with extended cognitive testing. These effects were only evident for the higher (100 μL) of the two doses investigated; raising the possibility that doses of this essential oil in excess of 100 μL would exert greater effects in terms of cognitive/mood enhancement.

## Figures and Tables

**Table 1:** Results from the initial screening of essential oils (receptor binding at a concentration of 0.1 mg/mL) and major constituents detected in each Mentha species by GC-MS analysis.

	*Mentha spicata*			*Mentha* × *piperita*		
**Essential Oil Number**	1	2	3	4	5	6
GABA_A_ receptor % displacement (^3^H) flunitrazepam	35 ± 3	25 ± 2	42 ± 5	42 ± 3	25 ± 2	15 ±2
Nicotinic receptor % displacement (^3^H) nicotine	30 ± 3	25 ± 4	21 ± 8	52 ± 5	50 ± 5	49 ± 1
NMDA receptor % displacement (^3^H) MK801	0 ± 3	0 ± 2	5 ± 4	0 ± 2	10 ± 3	5 ± 1
AChE Inhibition %	79.45	66.21	70.78	70.09	73.97	73.74
Major constituents detected in each sample by GC-MS						
% Limonene	20.8	20.8	18.4	2.3	2.4	2.0
% Carvone	61.9	57.5	58.8	0.1	0.1	0.1
% Menthone	0.4	1.7	0.1	24.9	24.7	24.2
% Menthol	0.5	2.8	0.80	36.7	32.5	34.7

**Table 2:** Cognitive Demand Battery pre-dose baseline (raw) data and “% change from baseline” data for the 1 h, 3 h and 6 h assessments. Serial 3 s/7 s errors post-dose scores are “change from baseline” (due to potential zero scores). All data shown are average scores across the 4 repetitions of each task during each assessment. Scores are mean (+ SEM).

		**Assessment**							
**TASK**	**Treatment**	**Baseline**		**1 h**		**3 h**		**6 h**	
Mental Fatigue (% VAS)	100 μL	61.81	4.08	11.46	5.32	10.16	6.40	21.42	7.92
	50 μL	58.10	3.92	12.43	7.26	17.70	7.55	26.28	7.73
	placebo	57.53	3.86	9.13	5.54	23.16	6.21	24.63	5.78
RVIP (% correct)	100 μL	51.05	4.55	2.78	4.01	−8.29	2.51	−9.77	2.91
	50 μL	57.19	4.01	−4.47	3.27	−13.07	3.37	−13.4	3.66
	placebo	54.43	3.72	−11.96	4.30	−15.73	3.23	−18.2	3.32
RVIP (speed—ms)	100 μL	477.11	9.68	−2.21	0.97	−2.97	1.13	−2.40	1.43
	50 μL	474.48	11.47	−0.13	0.66	−0.45	0.59	−1.04	0.99
	placebo	484.23	11.67	−2.88	0.80	−1.95	0.95	−2.53	1.07
Serial 7 s (number correct)	100 μL	29.17	2.24	6.22	2.18	9.90	4.29	8.34	3.52
	50 μL	29.11	2.55	4.75	3.00	10.11	3.15	8.21	4.18
	placebo	28.86	2.58	8.89	4.87	9.11	3.64	10.08	4.65
Serial 7 s (errors)	100 μL	2.24	0.33	0.05	0.26	0.51	0.32	−0.15	0.25
	50 μL	1.91	0.32	0.00	0.32	0.39	0.19	−0.10	0.38
	placebo	2.08	0.36	−0.28	0.27	0.20	0.33	−0.40	0.27
Serial 3 s (number correct)	100 μL	46.83	3.48	5.14	2.28	11.59	3.83	7.50	3.09
	50 μL	46.93	3.77	4.17	2.44	5.64	4.14	2.59	3.55
	placebo	46.61	3.50	3.96	2.26	1.83	2.27	5.14	2.61
Serial 3 s (errors)	100 μL	2.19	0.36	0.47	0.33	−0.33	0.41	0.13	0.22
	50 μL	2.44	0.43	−0.56	0.28	0.01	0.49	0.60	0.29
	placebo	1.60	0.26	−0.27	0.30	−0.48	0.37	−0.34	0.19

## Supplementary Materials

The following are available online at http://www.mdpi.com/2072-6643/10/8/1029/s1, Table S1: Percentage composition of compounds detected in the Mentha spicata and Mentha × piperita essential oils, obtained from the GC-MS total ion chromatograms, Table S2: Individual cognitive task performance: pre-dose baseline (raw) data and ‘% change from baseline’ data for the 1 h, 3 h and 6 h assessments. Immediate and Delayed Word Recall correct and error post-dose scores are ‘change from baseline’ (due to potential zero scores). Scores are mean (+ SEM), Table S3: Mood data: pre-dose baseline (raw) data and ‘% change from baseline’ data for the 1 h, 3 h and 6 h assessments. Appendix I: in vitro neuroprotective properties–method and results.
