# Supplementary material for: Volatile Terpenes and Brain Function: Investigation of the Cognitive and Mood Effects of Mentha × Piperita L. Essential Oil with In Vitro Properties Relevant to Central Nervous System Function
Source: Nutrients. 2018 Aug 7;10(8):1029. doi: 10.3390/nu10081029 (PMC6116079; doi:10.3390/nu10081029)
Supplement: Supplementary file 1 [file nutrients-10-01029-s001.pdf]

Volatile terpenes and brain function: Investigation of the cognitive and mood effects of Mentha × piperita L. essential oil with in vitro properties relevant to central nervous system function

Supplementary Tables:

Table S1. Percentage composition of compounds detected in the *Mentha spicata* and *Mentha* × *piperita* essential oils, obtained from the GC-MS total ion chromatograms.

**Compound**	**Retention time (min)**	***M. spicata* Oil 1 (%)**	***M. spicata* Oil 2 (%)**	***M. spicata* Oil 3 (%)**	***M. piperita* Oil 4 (%)**	***M. piperita* Oil 5 (%)**	***M. piperita* Oil 6 (%)**	**RI***	**RI [39]**
α-Thujene	11.4	0.08	0.01	0.06	0.02	0.04	0.02	900	924
α-Pinene	11.7	1.90	0.47	0.84	0.70	0.70	0.42	908	932
Camphene	12.5	0.29	0.03	0.03	0.01	0.05	0.05	924	946
Sabinene	13.6	0.96	0.12	0.60	0.27	0.47	0.32	948	969
β-Pinene	13.8	2.39	0.85	0.93	0.69	1.06	0.71	953	974
1-Octen-3-ol	13.9	Nd	Nd	Nd	0.02	0.06	0.05	957	974
3-Octanone	14.1	0.08	0.01	0.02	0.03	0.07	0.04	961	979
Myrcene	14.3	0.67	0.27	1.64	0.06	0.11	0.12	961	988
3-Octanol	14.8	1.06	0.29	0.39	0.09	0.18	0.17	974	988
p-Mentha-1(7),8-diene	15.0	0.15	0.03	0.12	0.01	0.03	0.02	980	1003
α-Phellandrene	15.1	Nd	Nd	Nd	0.01	0.01	0.02	983	1002
3-δ-Carene	15.4	0.07	0.02	Nd	0.01	0.01	0.01	990	1008
1,4-Cineole	15.6	0.01	0.01	Nd	Tr	Tr	Nd	993	1012
o-Cymene	16.0	0.61	0.79	0.42	0.25	0.92	0.54	1003	1022
Limonene	16.3	20.81	20.81	18.38	2.34	2.37	2.06	1010	1024
1,8-Cineole	16.4	1.01	0.81	2.06	5.80	5.59	5.34	1012	1026
(*Z*)-β-Ocimene	16.6	Nd	Nd	0.13	0.12	0.10	0.16	1015	1032
(*E*)-β-Ocimene	17.1	0.08	0.05	0.06	0.02	0.02	0.04	1026	1044
γ-Terpinene	17.6	0.10	0.09	0.32	0.14	0.08	0.22	1039	1054
cis-Sabinene hydrate	18.3	0.17	0.07	0.50	0.23	0.44	0.57	1054	1065

Terpinolene	19.0	0.09	0.04	0.11	0.06	0.04	0.08	1068	1086
p-Cymenene	19.2	0.05	0.03	0.05	0.01	0.01	0.02	1074	1089
Linalool	19.8	0.06	0.02	0.07	0.01	0.20	0.47	1086	1095
trans-Sabinene hydrate	19.9	0.01	0.01	0.07	0.07	0.13	Tr	1088	1098
3-Octanol acetate	20.7	0.01	0.02	0.12	0.02	0.04	0.04	1105	1120
trans-*p*-Mentha-2,8-dien-1-ol	20.8	0.01	0.01	0.04	0.01	0.02	0.01	1110	1119
cis-Limonene oxide	21.3	0.05	0.03	0.03	Tr	0.01	0.01	1121	1132
cis-*p*-Mentha-2,8-dien-1-ol	21.6	0.04	0.03	0.09	Tr	0.01	0.01	1126	1133
trans-Pinocarveol	21.8	Tr	0.01	0.04	Nd	Nd	Nd	1130	1135
trans-Sabinol	21.8	Nd	Nd	Nd	0.03	0.06	0.05	1131	1137
trans-Verbenol	22.0	0.02	Nd	0.03	Nd	Nd	Nd	1136	1140
Camphor	22.0	Tr	Tr	Nd	Tr	Nd	0.03	1137	1141
Isopulegol	22.2	0.01	0.21	0.01	0.12	0.12	0.13	1140	1145
Menthone	22.5	0.36	1.74	0.14	24.85	24.72	24.18	1146	1148
Iso-isopulegol	22.8	Tr	0.01	Nd	Nd	Nd	Nd	1152	1155
Menthofuran	22.9	Nd	Nd	Nd	2.91	2.19	3.41	1155	1159
Isomenthone	23.0	0.21	0.71	0.10	5.38	4.66	4.28	1156	1158
Neomenthol	23.2	0.05	0.39	0.16	3.39	3.64	3.95	1162	1161
Borneol	23.3	0.02	Nd	Tr	Nd	Nd	Nd	1165	1165
Menthol	23.6	0.47	2.79	0.80	36.69	32.49	34.71	1171	1167
Terpinen-4-ol	23.8	0.10	Tr	1.19	Nd	Nd	Nd	1173	1174
Isomenthol	24.1	Nd	0.10	Nd	0.51	0.74	0.79	1182	1179
Neoisomenthol	24.3	Nd	Nd	Nd	0.25	0.23	0.29	1187	1184
α-Terpineol	24.4	Nd	Nd	Nd	0.48	0.62	0.53	1189	1186
cis-Dihydro carvone	24.5	0.75	1.65	2.40	Nd	Nd	Nd	1191	1191

Dihydro carveol	24.7	0.56	1.54	Tr	Nd	Nd	Nd	1195	1192
trans-Dihydro carvone	24.8	0.24	0.42	0.38	Nd	Nd	Nd	1198	1200
trans-Carveol	25.7	0.10	0.47	0.52	Nd	Nd	Nd	1218	1215
Citronellol	26.1	Nd	Nd	Nd	0.04	0.04	0.03	1226	1223
cis-Carveol	26.4	0.08	0.58	0.24	Nd	Nd	Nd	1235	1226
Pulegone	26.5	Nd	Nd	Nd	1.42	1.47	1.63	1235	1233
Carvone	27.0	61.87	57.52	58.79	0.11	0.10	0.13	1240	1239
Piperitone	27.3	0.13	0.42	0.62	0.36	0.59	0.74	1253	1249
cis-Carvone oxide	27.6	0.12	0.10	0.21	Nd	Nd	Nd	1260	1259
Neomenthyl acetate	28.0	Nd	Nd	Nd	0.27	0.51	0.42	1268	1271
trans-Carvone oxide	28.2	Nd	Nd	0.18	Nd	Nd	Nd	1273	1273
Menthyl acetate	28.8	0.17	0.37	0.11	6.69	7.02	6.64	1287	1294
Isomenthyl acetate	29.5	Nd	Nd	Nd	0.17	0.37	0.27	1302	1304
Dihydro carveol acetate	29.5	0.04	0.43	0.03	Nd	Nd	Nd	1302	1306
Iso-dihydro carveol acetate	30.3	0.46	1.61	0.27	Nd	Nd	Nd	1320	1326
δ-Elemene	30.7	Nd	Nd	Nd	0.05	0.02	0.03	1328	1335
α-Cubebene	31.3	Nd	Nd	Nd	0.02	Tr	Tr	1343	1345
4aα,7α,7aα-Nepetalactone	31.5	Nd	Nd	Nd	0.06	0.32	0.17	1346	1357
Neoiso-dihydro carveol acetate	31.6	Nd	0.20	Nd	Nd	Nd	Nd	1349	1356
cis-Carvyl acetate	31.9	0.05	0.09	0.16	Nd	Nd	Nd	1355	1365
α-Ylangene	32.3	Nd	Nd	Nd	0.02	0.02	0.02	1365	1373
α-Copaene	32.6	Nd	Nd	Nd	0.05	0.06	0.09	1371	1374
β-Bourbonene	33.0	0.17	0.17	1.39	0.12	0.21	0.27	1379	1387
β-Elemene	33.2	0.07	0.09	0.14	0.07	0.13	0.11	1385	1389

(*Z*)-Caryophyllene	33.9	0.02	0.02	0.04	0.03	0.07	0.04	1399	1408
(*E*)-Caryophyllene	34.5	0.23	0.82	0.90	1.80	1.71	1.91	1414	1417
β-Copaene	35.0	0.03	0.04	0.30	0.05	0.07	0.09	1423	1430
α-*trans*-Bergamotene	35.2	Nd	Nd	Nd	Nd	0.03	0.01	1425	1432
(*Z*)-β-Farnesene	35.9	Nd	Nd	0.14	0.05	0.12	0.12	1444	1440
α-Humulene	36.1	0.01	0.08	0.13	0.11	0.11	0.10	1447	1452
Allo-aromadendrene	36.2	Nd	Nd	Nd	0.05	0.05	0.04	1451	1458
cis-Muurola-4(14),5-diene	36.6	0.01	0.03	0.09	0.02	0.02	0.02	1459	1465
γ-Muurolene	36.8	Nd	Nd	Nd	0.03	0.06	0.03	1465	1478
Germacrene D	37.1	Tr	0.03	0.32	0.36	0.02	0.28	1471	1484
Viridiflorene	37.5	Nd	Nd	Nd	0.03	0.05	0.03	1480	1496
Mint furanone[a]	37.8	Nd	Nd	Nd	Nd	0.33	Nd	1487	-
α-Muurolene	37.9	0.01	0.02	0.03	Nd	Nd	Nd	1488	1500
γ-Cadinene	38.5	Nd	Nd	Nd	0.02	0.04	0.02	1500	1513
δ-Cadinene	38.6	Tr	0.02	0.02	0.04	0.08	0.04	1504	1522
Spathulenol	41.0	Nd	Nd	Nd	0.01	0.11	0.03	1558	1577
Caryophyllene oxide	41.3	0.04	0.10	0.04	0.10	0.68	0.20	1563	1582
Viridiflorol	41.7	Nd	Nd	Nd	0.04	0.16	0.04	1574	1592

All compounds identified by comparing retention indices [RI] (calculated against an *n*-alkane series) and by comparing mass spectra with published data [39,40], except for [a]compounds identified by comparison with published mass spectra only [40]. *Experimental RI value. Nd: Not detected / below level of detection. Tr: < 0.01%.

Table S2.. Individual cognitive task performance: pre-dose baseline (raw) data and '% change from baseline' data for the 1 hr, 3 hr and 6 hr assessments. Immediate and Delayed Word Recall correct and error post-dose scores are 'change from baseline' (due to potential zero scores). Scores are mean (+ SEM).

				ASSESSMENT					
TASK	Treatment	Baseline		1 hr		3 hr		6 hr	
Corsi Blocks (span score)	100 µL	6.35	0.17	-0.20	2.57	1.18	2.68	-3.03	2.06
	50 µL	6.30	0.14	-0.73	3.37	-0.32	2.77	-6.64	2.63
	placebo	6.23	0.20	3.39	2.84	0.46	3.34	1.63	2.62
Choice Reaction Time (% correct)	100 µL	97.83	0.52	-0.83	0.77	-0.15	0.49	-0.60	0.58
	50 µL	96.87	0.87	0.20	0.74	0.02	0.77	0.36	0.62
	placebo	97.65	0.73	-0.70	0.40	-0.98	0.62	-0.86	0.65
Choice Reaction Time (speed - msecs)	100 µL	411.83	12.69	4.10	2.58	4.20	3.20	1.56	2.14
	50 µL	421.84	14.57	5.36	3.57	2.96	2.93	-0.56	1.81
	placebo	412.24	10.73	1.37	1.46	2.21	1.65	-1.33	1.53
Name-to-Faces (% correct)	100 µL	54.17	4.36	-9.67	7.23	8.86	16.01	0.28	11.05
	50 µL	61.55	3.56	-17.9	4.96	-22.4	6.19	-14.89	5.41
	placebo	57.95	3.58	-8.59	9.69	-9.72	8.01	-17.46	6.25
Numeric Working Memory (% correct)	100 µL	94.44	0.90	0.74	0.82	0.49	0.90	-0.70	0.94
	50 µL	94.49	0.84	0.08	0.73	-0.11	0.83	-0.61	1.00
	placebo	94.93	0.77	-0.36	1.03	-0.64	1.01	-1.75	0.89
Numeric Working Memory (speed - msecs)	100 µL	722.14	40.28	-1.00	2.29	-0.70	2.41	-1.13	2.23
	50 µL	693.32	33.54	2.03	2.48	-0.71	2.74	-1.25	2.01
	placebo	684.89	27.22	1.15	1.99	-2.16	1.90	1.14	3.06
Peg and Ball (completion time – msecs)	100 µL	6592.0	471.09	-3.57	2.81	-8.20	1.97	-6.06	2.27
	50 µL	6623.4	354.29	-2.71	2.54	-9.68	3.06	-10.32	2.43
	placebo	6685.5	371.52	-5.28	2.17	-11.3	1.89	-9.37	2.45
Peg and Ball (thinking time – msecs)	100 µL	1804.2	153.23	-4.65	5.98	-8.68	5.20	-11.53	5.04
	50 µL	1919.7	182.45	-8.68	5.35	-9.89	6.56	-14.20	5.18
	placebo	1773.0	127.22	-5.90	5.64	-12.7	4.60	-11.86	5.00
Picture Recognition (% correct)	100 µL	89.42	2.38	-2.39	2.67	-7.59	2.08	-8.59	2.67
	50 µL	89.86	1.99	-7.76	2.60	-7.75	1.94	-6.77	2.34
	placebo	85.65	2.24	2.28	1.91	-1.61	2.59	-5.99	3.25
Picture Recognition (speed - msecs)	100 µL	759.46	24.46	2.33	2.06	3.92	1.96	5.76	3.91
	50 µL	785.90	34.59	-1.85	2.36	-1.71	3.98	-0.79	2.92
	placebo	777.22	44.22	-2.54	2.27	-1.52	2.56	-2.57	2.80
RVIP (% correct)	100 µL	60.34	4.12	-3.67	5.33	-8.67	4.47	-14.55	4.96
	50 µL	61.36	3.86	-5.78	5.17	-10.6	4.30	-10.66	4.97
	placebo	60.00	3.47	-5.22	5.24	-14.1	4.09	-11.85	3.41
RVIP (speed - msecs)	100 µL	459.13	9.52	-1.02	1.22	-0.25	1.60	0.58	1.69
	50 µL	472.24	13.23	-0.18	1.51	-1.99	1.53	-2.72	1.19
	placebo	476.75	13.51	-1.37	1.09	-3.43	1.46	-4.20	1.54
Word Recognition (% correct)	100 µL	77.10	2.44	-4.28	3.45	-1.79	3.51	-5.43	3.41
	50 µL	75.36	2.73	-0.85	3.55	-1.25	3.98	1.63	4.48
	placebo	79.42	1.66	-8.33	2.69	-9.45	2.48	-11.38	3.18
Word Recognition (speed – msecs)	100 µL	770.09	33.58	7.41	3.27	0.07	2.68	1.83	2.93
	50 µL	743.33	29.70	4.38	3.18	2.37	3.48	1.48	2.34
	placebo	770.27	32.12	-1.57	2.52	-4.60	3.15	-1.89	3.65
Immediate Word Recall (number)	100 µL	7.61	0.44	0.30	0.28	-0.09	0.23	0.04	0.23
	50 µL	7.70	0.49	0.30	0.28	0.00	0.23	0.09	0.23
	placebo	7.61	0.44	-0.09	0.15	-0.04	0.18	0.04	0.21
Delayed Word Recall (number)	100 µL	5.07	0.53	-2.04	0.46	-1.91	0.49	-2.74	0.69
	50 µL	5.34	0.68	-1.78	0.65	-2.17	0.53	-2.22	0.43
	placebo	5.20	0.58	-1.37	0.42	-2.59	0.47	-3.13	0.64

Table S3. Mood data: pre-dose baseline (raw) data and '% change from baseline' data for the 1 hr, 3 hr and 6 hr assessments. Scores are mean (+ SEM).

		ASSESSMENT							
TASK	Treatment	Baseline		1 hr		3 hr		6 hr	
State-Trait	100 µL	32.96	1.55	0.39	0.69	0.87	0.72	0.57	0.99
Anxiety inventory	50 µL	33.39	1.71	1.43	0.70	0.78	0.63	0.83	0.66
(State)	placebo	33.04	1.72	1.13	0.40	1.78	0.59	0.83	0.91
Bond-Lader VAS	100 µL	61.99	2.62	2.30	4.53	-0.20	4.63	-5.33	4.82
Alert	50 µL	61.68	3.26	1.77	5.28	-2.73	6.59	-7.33	6.10
(% along lines)	placebo	61.61	3.30	0.77	3.11	-2.48	3.22	-0.42	4.22
Bond-Lader VAS	100 µL	67.93	2.58	1.90	2.16	-0.91	2.30	-0.71	2.00
Content	50 µL	64.42	2.85	2.27	1.87	-2.43	2.49	-2.20	2.77
(% along lines)	placebo	67.55	2.29	-3.19	2.27	-4.93	2.44	-5.68	4.07
Bond-Lader VAS	100 µL	65.67	2.87	3.39	4.62	-4.52	3.55	-6.02	4.79
Calm	50 µL	63.20	2.58	-6.41	4.15	-2.62	4.23	-13.0	4.22
(% along lines)	placebo	63.81	2.51	-1.16	5.99	-1.89	4.67	-2.06	6.71

Appendix I. Neuroprotective properties – Method and results

CAD cell culture

CAD (Cath.-a-differentiated) cultures were grown at 37°C and in 5% CO2 on 75 cm^2 tissue culture flasks (Sarstedt, Newton, NC) in Dulbecco's modified Eagles' medium DMEM/F-12 Media - GlutaMAX™-I (GIBCO, Grand Island, NY), supplemented with 10% foetal bovine serum (FBS; Sigma, St. Louis, MO, USA). Cells were passaged every 6–7 days at a 1:4 dilution.

Survival of CAD cells was assessed using an MTT assay after 1 h pre-treatment with *M piperita* (0.1 mg/ml (w/v)) followed by 24 h post-treatment with 250 µM H_2O_2. All the treatments was performed using n = 6 replicates. The tetrazolium dye MTT (Sigma, UK) was used to assess cell viability as described by Abuhamdah *et al.*, (2015). 50µl Phosphate buffered saline (PBS) (PBS) (136.9 mm 2.68 mm KCl, 4.3 mm Na_2HPO_4, 1.4 mm KH_2PO_4, pH 7.4) containing a final concentration of 5 mg/ml MTT was added to the cultures and incubated at 37°C and in 5% CO_2 for 2.5 h. The MTT containing medium was then removed and, the surface of the wells was rinsed gently with 300 µl PBS before the application of 250 µL isopropanol. The optical density of 100 µL samples was spectrophotometrically read at 595 nm. (Thermo Lab systems Multiskan Ascent, V1.3).

M piperita essential oil (Sample 4) was not neurotoxic up to 0.1mg/ml (not shown). Using this concentration, a modest but significant degree of neuroprotection was observed Vs 250 µM H_2O_2. (Figure 1), consistent with previous work.

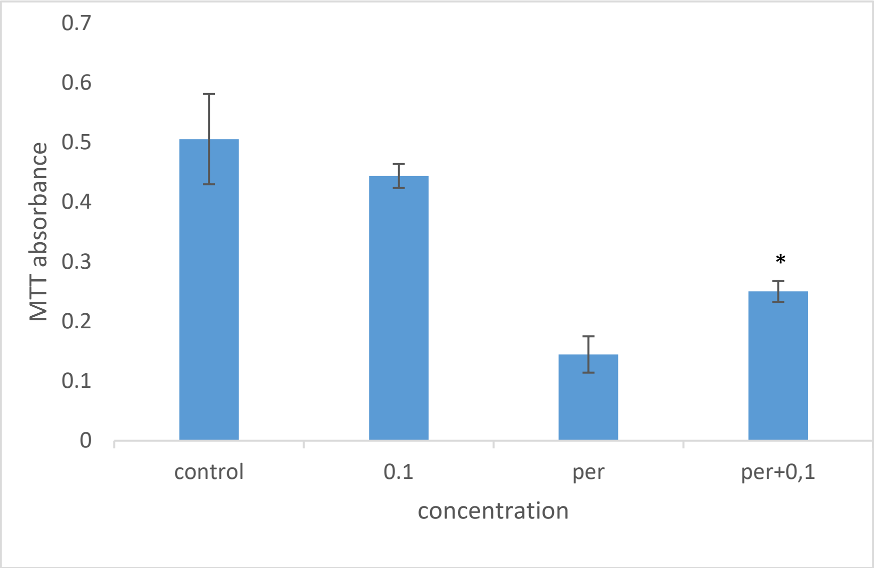

Figure 4. Neuroprotective effects of *M. Piperita* essential oil. Cell viability (MTT absorbance) was assayed following exposure to HBS (control), 1mg/ml *M .piperita* essential oil alone, 250 µM hydrogen peroxide or 1mg/ml *M .piperita* essential oil plus 250 µM hydrogen peroxide for 24 hours. 250 µM hydrogen peroxide elicited a robust reduction in viability (75%). Partial protection of CAD neuronal cells was observed with *M .piperita* essential oil (0.1 mg/ml). * $p < 0.05$ Vs hydrogen peroxide alone. Representative data (6 replicate samples) from n = 3 individual experiments.
